# Measuring Blood Pulse Wave Velocity with Bioimpedance in Different Age Groups

**DOI:** 10.3390/s19040850

**Published:** 2019-02-19

**Authors:** Shafa Aria, Yassine Elfarri, Marius Elvegård, Adam Gottfridsson, Halvor S. Grønaas, Sigve Harang, Anders Jansen, Thomas Emil Rolland Madland, Ivar Bruvik Martins, Marius Wilhelm Olstad, Tommy Lee Ryan, Anwar Nazih Shaban, Øyvind Løken Svenningsen, Andre Djupvik Sørensen, Emil Holm Ulvestad, Ole Martin Vister, Morten Bratgjerd Øvergaard, Håvard Kalvøy, Fred Johan Pettersen, Hans Henrik Odland, Vegard Munkeby Joten, Øyvind Grannes Martinsen, Christian Tronstad, Ole Elvebakk, Ørjan Grøttem Martinsen

**Affiliations:** 1 Department of Physics, University of Oslo, 0372 Oslo, Norway; shafa.aria@fys.uio.no (S.A.); yelfarri@gmail.com (Y.E.); marius.elvegard@me.com (M.E.); adamgottfridsson@gmail.com (A.G.); halvorg@gmail.com (H.S.G.); sigvesh@student.matnat.uio.no (S.H.); andersj1977@gmail.com (A.J.); thomas.madland@gmail.com (T.E.R.M.); ivarbm@gmail.com (I.B.M.); marolst@hotmail.com (M.W.O.); tommy.lee.ryan@gmail.com (T.L.R.); anwarns@student.uv.uio.no (A.N.S.); oyvindls@student.matnat.uio.no (Ø.L.S.); andredsor@hotmail.com (A.D.S.); emil.ulvestad@gmail.com (E.H.U.); ole_m_v@hotmail.com (O.M.V.); morten.overgaard@gmail.com (M.B.Ø.); 2 Department of Clinical and Biomedical Engineering, Oslo University Hospital, 0372 Oslo, Norway; hkalvoy@ous-hf.no (H.K.); fjp.medtek@aerogadget.net (F.J.P.); christian.tronstad@gmail.com (C.T.); ole.elvebakk@gmail.com (O.E.); 3 Department of Physics, University of Oslo, 0316 Oslo, Norway; v.m.joten@fys.uio.no (V.M.J.); oyvindgm@fys.uio.no (Ø.G.M.); 4 Department of Cardiology, Oslo University Hospital, 0372 Oslo, Norway; hanshenrikodland@gmail.com

**Keywords:** impedance cardiography, pulse wave velocity, arteriosclerosis, bioimpedance

## Abstract

In this project, we have studied the use of electrical impedance cardiography as a possible method for measuring blood pulse wave velocity, and hence be an aid in the assessment of the degree of arteriosclerosis. Using two different four-electrode setups, we measured the timing of the systolic pulse at two locations, the upper arm and the thorax, and found that the pulse wave velocity was in general higher in older volunteers and furthermore that it was also more heart rate dependent for older subjects. We attribute this to the fact that the degree of arteriosclerosis typically increases with age and that stiffening of the arterial wall will make the arteries less able to comply with increased heart rate (and corresponding blood pressure), without leading to increased pulse wave velocity. In view of these findings, we conclude that impedance cardiography seems to be well suited and practical for pulse wave velocity measurements and possibly for the assessment of the degree of arteriosclerosis. However, further studies are needed for comparison between this approach and reference methods for pulse wave velocity and assessment of arteriosclerosis before any firm conclusions can be drawn.

## 1. Introduction

Arteriosclerosis is one of the major determinants of cardiovascular risk. Numerous studies have attempted to quantify arteriosclerosis by assessing the arterial stiffness, for instance by assessing the pulse wave velocity (PWV) of the systolic blood pressure pulse in the major arteries [1,2,3,4]. PWV is calculated by taking the ratio of the distance between two positions at the body and the time difference between the occurrences of the systolic pressure pulse at these locations. For an exact measurement of the PWV, invasive pressure pulse measurements are indicated, but these are not suitable for widespread applications [5]. Many non-invasive techniques have been used, but difficulties in the determination of the distance and time difference make these techniques vulnerable to dispute [3]. An important drawback is that the systolic blood pressure pulse cannot be recorded non-invasively at the point of its genesis: The aortic root. This leads to approaches, such as where the pulse at the carotid artery is recorded and an estimated correction factor for the systematic error in the location is introduced [2].

Different methodologies have been implemented in commercially available devices for non-invasive regional PWV measurement. Current methods are based on electromechanical solutions with direct contact with the patient’s tissues at the artery site, which may distort the waveform [6] and be uncomfortable to the patient during measurement [7]. Most techniques assess the PWV by analyzing the timing characteristics of the pressure wave, distension wave or the flow wave. Examples of commercially available devices are the PulsePen^®^ (combining applanation tonometry with an electrocardiogram, or ECG) [8], the Complior^®^ (employing piezoelectric pressure mechanotransducers) [9], the SphygmoCor^®^ (sequential applanation tonometry and ECG) [10] and the Arteriograph^®^ (oscillometric pressure curve analysis) [11]. For more information on current techniques for PWV assessment, see References [6] or [12]. With respect to newer techniques, Pereira et al. [6] mention optical solutions for overcoming the problem of capturing pulse waveforms deeper into the skin.

A more convenient, non-invasive technique may be found in electrical impedance cardiography by recording an impedance cardiogram (ICG) [13]. The ICG measured at the thorax shows a marked pulse, the C-wave or ejection wave, at the moment of systolic ejection. Van Eijnatten et al. showed in 2014 that the peak of this wave coincides with the moment of maximum diameter of the aortic arch during the systolic phase of the cardiac cycle, evaluated by echocardiography [14]. Measuring ECG together with ICG, the initial systolic time interval (ISTI) can be derived, indicating the time delay between electrical and mechanical pumping activity of the heart [15]. This pulse travels through the arterial tree and can be observed non-invasively in the whole body regardless of the depth of the artery. This means that bioimpedance measurements may provide an alternative technique for determining the PWV, also at positions where the blood pressure pulse cannot easily be observed by other non-invasive techniques. It is also possible that this technique provides additional, independent information. It was the purpose of this study to investigate the feasibility of this novel technique of electrical bioimpedance and associated signal parameterization for non-invasive PWV assessment in humans by determining the timing of the systolic pulse at two locations, the upper arm and the thorax. As an early validation of the technique, it was investigated whether these timings differ between age groups of subjects and varies with different physiological states (by varying heart rate) in agreement with established knowledge and the scientific literature on PWV in humans. In later studies, the PWV, measured by electrical bioimpedance, can be compared to those obtained from pressure pulse techniques measured at the same locations. In this study, the bioimpedance-derived timing difference (ΔISTI) should satisfy the following two hypotheses in order to be in agreement with the established knowledge on PWV:

1. The ΔISTI is lower in older subjects (above 60) compared to younger subjects (below 30) from a healthy population.
2. The ΔISTI decreases with increasing heart rate in older subjects (above 60).

## 2. Materials and Methods

### 2.1. Experimental Design

Twenty healthy volunteers in two age groups were recruited to this study (see Table 1). Group 1 consisted of five female and five male participants in the age 20–29 years and group 2 consisted of five female and five male participants in the age 60–79 years. They all gave informed consent to participate.

**Figure 1:**
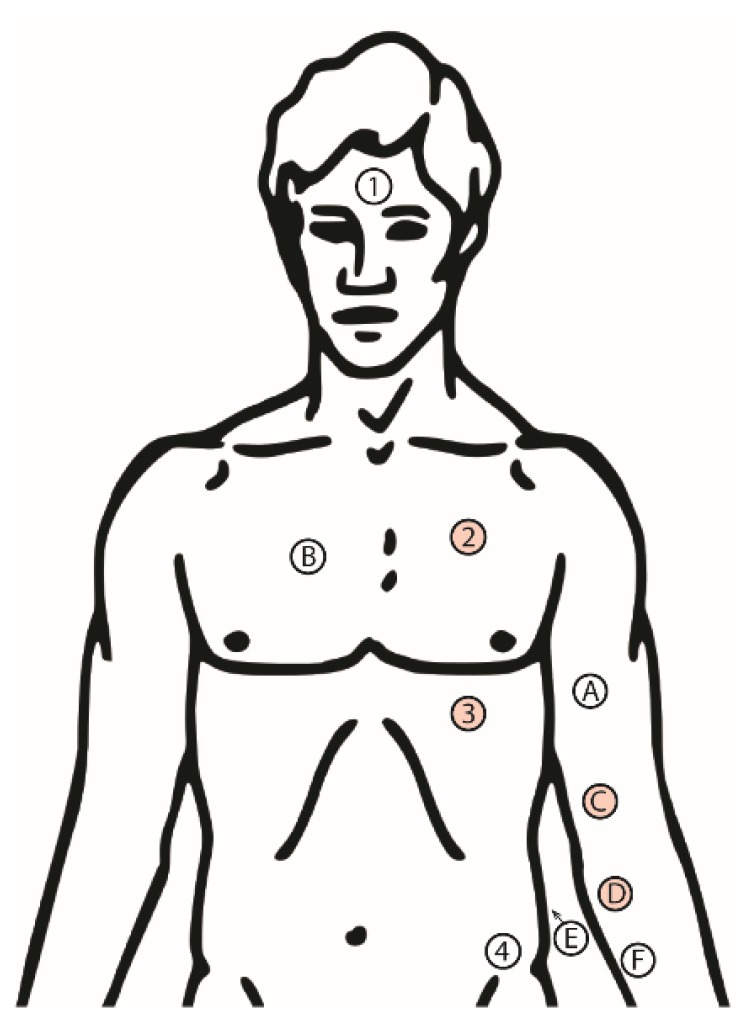
Placement of electrodes for electrocardiogram (ECG) and impedance cardiogram (ICG) measurement. ECG was measured between electrodes B and E. Thorax ICG was measured by electrodes 1–4, picking up impedance changes between electrodes 2–3. Upper arm ICG was measured by electrodes A, C, D and F, picking up impedance changes between electrodes C and D.

The measurements were performed in a laboratory at the University of Oslo and the volunteers were mainly recruited among students and employees. Ten Ambu^®^ Blue Sensor ECG electrodes (type Q-00-S) were attached on the upper body of the test subjects after washing each skin site with alcohol for improved electrical contact (mainly by removing sebum from the skin surface). Figure 1 shows the skin sites used. 

Electrodes 1–4 were connected to an ICG bioimpedance instrument operating at 64 kHz in such a way that a constant current of 300 µA rms was injected through electrodes 1 and 4, while the differential voltage was picked up between electrodes 2 and 3. This enabled the calculation of the transfer impedance for this four-electrode setup [16]. The instrument, which was described by Meier et al. [15], also measures the ECG signal between electrodes 2 and 3, the derivative of the measured transfer impedance, dZ/dt, and the ECG signal at the output connector. However, the ECG signal from this instrument was not used in this study. A similar instrument was used on the arm to measure the transfer impedance with electrodes A and F as the current input and electrodes C and D for the differential voltage pick-up. This instrument used separate electrodes B and E for ECG monitoring. The output from the two instruments was connected to a National Instruments Data Acquisition Card type USB-6356 and data were logged in a custom-made National Instruments LabVIEW^®^ program. The DAQ card had a synchronous sampling of the analog channels to avoid any synchronization issues.

The volunteers were then first asked to pedal at a moderate speed for one minute on a stationary bicycle with low mechanical resistance on the wheel. Then they rested on the bicycle for one minute while the recordings of the ICG and ECG were performed. The mechanical resistance on the bicycle wheel was then increased to an intermediate level and the volunteers now pedaled for three minutes at a somewhat higher speed. Another recording period of one minute followed this before the volunteers were asked to pedal one last time for three minutes, with high mechanical resistance, and at the maximum speed that they could tolerate without too much discomfort. A third recording period of one minute followed this. 

The first recording period in the protocol was regarded as a training part for the volunteers, enabling them to adapt to the activity of cycling and subsequent relatively motionless sitting for optimal quality of the recorded data. Hence, only recording periods 2 and 3 were used for data analysis.

**Figure 2:**
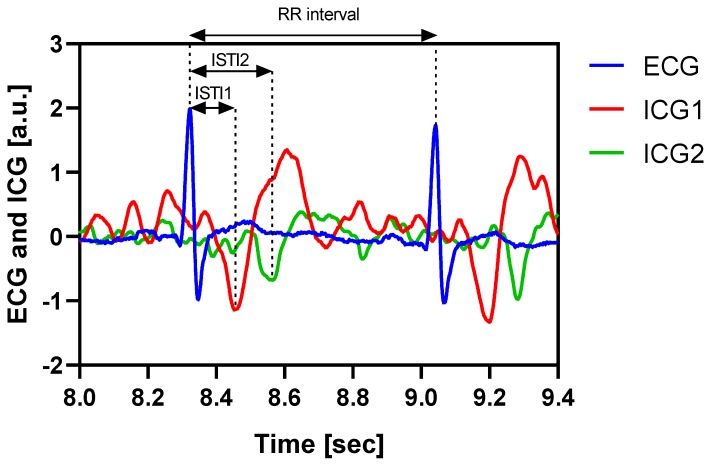
Example measurement showing ECG (blue), ICG across heart (red), and ICG on left arm (green). The ICG complexes are the calculated dZ/dt signals from the transfer impedance measurements. The bioimpedance-derived timing difference (ISTI) at the thorax (ISTI1) and upper arm (ISTI2) are marked in arrows, along with the RR interval.

Example measurements of the ECG and two ICG (meaning dZ/dt) recordings are shown in Figure 2. 

All data files were analyzed using Matlab R2016b. Each dataset was plotted and the R-points in the ECG and the C-points in the ICG were all marked manually using the Matlab ginput function (graphical input from mouse or cursor). Subsequently, the initial systolic time intervals for the ICG over the heart and arm (ISTI1 and ISTI2, respectively), were calculated for about every second heartbeat (actually for three heartbeats in every 5-s interval). The ISTI is the time from the R-peak of the ECG to the corresponding C-peak of the ICG. The difference between the ISTI2 and ISTI1 (ΔISTI) was used to indicate the pulse travel time from the thorax to the upper arm. Although the mathematical determination of ΔISTI is independent of the R-points and the ECG signal, the ECG signal is very useful as a reference for locating the correct troughs as C-points in the ICG signal. The corresponding RR intervals, i.e., the time between the two consecutive R peaks in the ECG, were also calculated. In order to calculate the PWV, we measured the distance between the suprasternal notch and the midpoint, between the voltage pick-up electrodes on the arm on each individual test subject. This distance was assumed to be a good approximation to the distance between the anatomical measurement focus of the two ICG systems [17]. This value was then divided by the ΔISTI to find the PWV. The inverse of the PWV, i.e., the ΔISTI divided by the above-mentioned anatomical distance, is hereafter called the “distance corrected ΔISTI”, or ΔISTIc. 

### 2.2. Statistical Analysis

In order to test whether the ΔISTIc differs between the two age groups, and also whether the ΔISTIc depends on the RR interval, a linear mixed effects (LME) model was employed. The ΔISTIc was modeled as a function of the Age Group and RR (with individual slopes for each subject) including an interaction term between the two effects (Age Group and RR) in order to test whether the Age Group difference in the ΔISTIc depended on the RR. 

Due to a significant interaction between the Age Group and RR, the test for age group differences in the ΔISTIc was done at two selected RR intervals: 500 ms and 1000 ms. Linear regression was used to estimate the ΔISTIc value at exactly these RR intervals for all subjects, as done by van Eijnatten et al. [14]. The ΔISTIc (RR = 500 ms) and ΔISTIc (RR = 1000 ms) were then statistically compared between the two age groups using the unpaired Student t-test (or the Mann–Whitney Rank Sum test for observations not following a normal distribution based on the Shapiro–Wilk test). 

In order to test the effect of the RR on the ΔISTIc for both age groups separately, an LME model for the ΔISTIc, as a function of the RR and Age (of the subject, with individual slopes for each subject), including the RR*Age interaction, was first employed for both of the age groups. Finding no significant interaction between the RR and Age (within both the young and old age groups), the ΔISTIc–RR relationship was determined by a model using only the RR and Age as the main effects, providing an estimate for the ΔISTIc–RR relation, controlling for any effect of age. 

A test for difference in the distributions of the RR values between the two age groups, based on the two-sample Kolmogorov–Smirnov test, was also done in order to evaluate whether any difference in the RR levels from the measurements on the younger and older subjects could inflict the results. This test was done by an LME model for the RR as a function of the Age Group (with random intercept), finding no significant effect of the Age Group on the RR, indicating that the levels of heart rate during the experiments would not confound the between-group comparisons.

The statistical computation was performed using Matlab R2016b and Sigmaplot v11.0.

### 2.3. Ethical Approval

The research related to human use has been complied with all the relevant national regulations (including the Regional Committees for Medical and Health Research Ethics), institutional policies and in accordance with the tenets of the Helsinki Declaration. 

## 3. Results

### 3.1. Effects of Age and RR Interval on ΔISTIc

**Figure 3:**
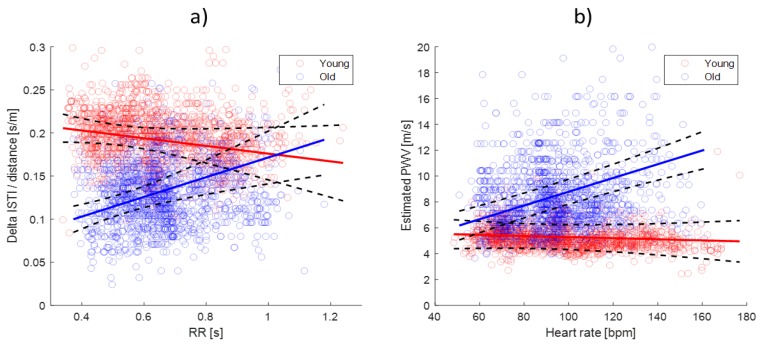
(**a**) The relation between ΔISTIc (distance-corrected ΔISTI, as explained earlier) and RR for the old (blue) and young (red) subjects. (**b**) The relation between the variables transformed to estimated PWV and heart rate. All observations pooled from the old and young age groups are plotted in blue and red circles respectively. Regression lines for the two groups from the linear mixed model are added to the plot (solid lines) with confidence intervals in black. Few outliers up to ΔISTIc = 0.41 s/m and PWV = 41.7 m/s are not shown for graph visibility.

The linear mixed effects model revealed that there was a statistically significant interaction between the Age Group and RR interval (*p* < 0.001). Consequently, an overall age-related difference in the ΔISTIc could not be determined, because this difference depended on the heart rate. Figure 3 shows the relation between the ΔISTIc and RR for both age groups, indicating that there was a difference between the age groups at a lower RR (higher heart rates), which diminished as the RR increased towards 1 s (60 beats per minute).

### 3.2. ΔISTIc Difference between Young and Older Subjects at High and Low Heart Rate

Due to the RR dependency on the age-related ΔISTIc difference, the ΔISTIc was compared between the age groups at two selected heart rates: RR = 500 ms and RR = 1000 ms. At RR = 500 ms, the young subjects had a higher ΔISTIc than the older subjects did (mean difference of 0.084 s/m, 95% CI of 0.065 to 0.10, *p* < 0.001 based on an unpaired Student’s t-test). At 1000 ms, there was no significant difference in the ΔISTIc between the two age groups (*p* = 0.43, Mann–Whitney Rank Sum test). 

### 3.3. ΔISTIc Relation to RR for Young and Older Subjects

For the young subjects, the ΔISTIc was independent on the RR (*p* = 0.09) and Age (*p* = 0.60). For the old subjects, the ΔISTIc was significantly dependent on the RR (*p* < 0.001) with a slope of 0.12 s/m of the ΔISTIc per second of the RR (SE = 0.035), and no significant effect of Age within the group. This relation was also estimated for the PWV (1/ΔISTIc) vs. heart rate (1/RR∙60), giving a slightly negative but not significant relationship for the young group (-0.004 m/s, SE = 0.003 per increase in beats per minute), and a significant (*p* < 0.001) positive relation for the old group with a PWV increase of 0.053 m/s (SE = 0.011) per increase in beats per minute. This relationship is shown visually in Figure 3b.

### 3.4. Example Recordings

**Figure 4:**
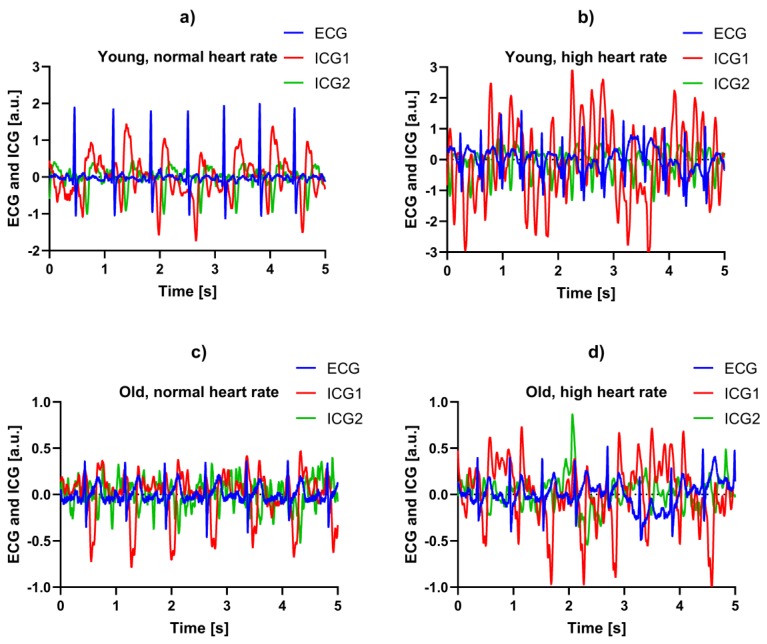
Examples of ECG and ICG recordings from different participants from both age groups during low and high heart rate. The people were sitting still on a stationary bike during the recordings. (**a**) Young participant in relaxed state, (**b**) young participant after an intense period of pedaling, (**c**) older participant in relaxed state, (**d**) older participant after an intense period of pedaling.

Selected raw data examples of the recordings are shown in Figure 4 for old and young participants at low and high heart rates. In Figure 4a,c the participants had only pedaled for one minute with low intensity and mechanical resistance, while in Figure 4b,d the participants had been through all three intervals, and hence just pedaled for three minutes at high intensity and mechanical resistance. As shown for example, in Figure 4d, the signal quality of the ICG recording was not always good, requiring careful inspection and comparison with the ECG as a reference for correct identification of the timing parameters. It could also be seen that the shapes of the ICG waveforms are different from subject to subject, but that the C-wave (first through after the onset of the ECG T-wave) was more similar between individuals and states.

It was shown that the bioimpedance technique could be used practically to estimate the PWV non-invasively in humans by determining the timing of the systolic pulse at the chest and upper arm, estimated in meters per second by the inverse of the ΔISTIc parameter. A significantly higher ΔISTIc was found for the younger subjects than for the older, particularly at a low RR (high heart rate) values. The difference diminished as the RR interval increased. A plausible explanation for this was the general increase of arteriosclerosis, with age leading to reduced elasticity of the arterial wall and hence increased pulse wave velocity. 

## 4. Discussion

Both hypotheses were supported by the data in this study, implying that the bioimpedance-derived ΔISTI parameter and the respective PVW estimate is in agreement with the established knowledge on the PVW in a healthy population. The older subjects had a significant trend in the ΔISTIc with the RR, while the younger subjects did not. A possible explanation for this is that an increase in heart rate is typically accompanied by an increase in blood pressure. The higher the elasticity of the arterial wall, the more the arteries can compensate for this increased pressure without resulting in a higher pulse wave velocity [18]. Hence, in older subjects with presumed increased arterial stiffness, the PWV will increase with increasing heart rate, due to the accompanying increase in blood pressure. As shown visually in Figure 4, the distance between the troughs of the two ICG C-waves was narrowed (indicating a higher PWV due to stiffer arteries) for the older participants, in particular for the high heart rate condition. This relation between the PWV and heart rate was in agreement with Lantelme et al. [19], who reported increasing carotid-femoral PWV with increasing heart rates in a population of elderly (mean age 77.8) patients after pacemaker implantation, and Tan et al. [20], who also reported an increase in carotid-femoral PWV with increasing heart rate in older patients (40–93), even when correcting for blood pressure dependency of the PWV. Xiao et al. [21] performed a simulation study on the relationship between the PWV and heart rate by a transmission line model of the arterial tree, exploring a viscoelastic mechanism for the influence of heart rate on arterial stiffness. The study demonstrated a possible mechanism on how the PWV depends on the frequency-dependency of the arterial wall viscoelasticity, being lower in younger elastic arteries, corresponding with a lower heart rate influence on the PWV. Although not statistically significant, the results (Figure 3) indicated a possible slight negative relationship between the ΔISTIc and RR for the young subjects, which was a possible outcome for arteries with the lowest frequency dependence of the arterial wall elasticity in the simulation study by Xiao et al. [21].

The average estimated PWV for both age groups was similar to the values reported in Tomiyama et al. [22], based on a large population of participants from 25–87 years of age. For the heart-brachial measurement, the mean PWV was around 6.5 m/s at 70 years of age, and around 5.0 m/s at 25 years of age, at a heart rate around 65 bpm. Corresponding values from our study were around 6.9 m/s for the old group (mean age 68.2 years) and around 5.5 m/s for the young group (mean age 24.6). The estimated PWV dependency on heart rate from our study was similar to the estimate from Lantelme et al. [19], where the average carotid-femoral PVW vs. heart rate slope was 0.04 m/s per beat per minute (vs. 0.053 in our study). 

Although the ECG could be considered redundant mathematically in deriving the ΔISTI, ΔISTIc, and estimated PWV, the ECG signal was very helpful in locating the correct timing points (C-points) in the ICG signal. In addition, the significant PWV dependency on heart rate suggests that the ECG also was necessary to include for heart rate correction in the determination of individual PWV characteristics. The location of timing points was done manually in this study in order to optimize the data quality, but an automated analysis could be developed in the future using more advanced signal processing methods.

This study was limited with respect to assessing the accuracy of the PWV, as no reference method was included. The sample size was low with respect to estimating the PWV mean values for the populations that the samples were drawn from. However, this study demonstrated the feasibility of using the bioimpedance technique for estimating the PWV by the ΔISTIc, derived from the ECG and ICG measurement at the thorax and upper arm, and the measurements obtained were in agreement with results from larger studies using other measurement techniques. 

We believe that the results are encouraging and call for further development and validation of the technique. This includes developing integrated instrumentation tailored for the usage, optimizing the ICG signal quality and the development of robust automatic signal processing methods for accurate ΔISTIc derivation. A multichannel real-time bioimpedance measurement device specifically designed for pulse wave analysis was recently presented by Kusche et al. [23], demonstrating good signal to noise ratio for the acquisition of miniscule bioimpedance signals (such as the pulsatile changes at the arm). For validation of the PWV estimation by the bioimpedance technique, future studies are necessary on a larger sample including measurements with a reference technique such as ultrasound.

## 5. Conclusions

In view of the findings of this study, we conclude that the technique of impedance cardiography with two four-electrode setups and associated timing seems to be well suited and practical for PWV measurement and hence, possibly for the assessment of the degree of arteriosclerosis. However, further studies are needed for comparison between this approach and reference methods for the PWV, and assessment of arteriosclerosis, before any firm conclusions can be drawn.

## Figures and Tables

**Table 1:** Data for the test subjects. Values are in mean ± SD.

Group	Male	Female	Age (years)	Height (cm)	Weight (kg)
Younger	5	5	24.6 ± 2.3	176.4 ± 7.2	71.3 ± 13.6
Older	5	5	68.2 ± 5.9	174.6 ± 8.7	75.7 ± 8.7
